# α-Tocopherol Protects Lipopolysaccharide-Activated BV2 Microglia

**DOI:** 10.3390/molecules28083340

**Published:** 2023-04-10

**Authors:** Maria Ester La Torre, Antonia Cianciulli, Vincenzo Monda, Marcellino Monda, Francesca Martina Filannino, Laura Antonucci, Anna Valenzano, Giuseppe Cibelli, Chiara Porro, Giovanni Messina, Maria Antonietta Panaro, Antonietta Messina, Rita Polito

**Affiliations:** 1 Department of Clinical and Experimental Medicine, University of Foggia, 71122 Foggia, Italy; ester.latorre@unifg.it (M.E.L.T.); francesca.filannino@unifg.it (F.M.F.); laura.antonucci@unifg.it (L.A.); anna.valenzano@unifg.it (A.V.); giuseppe.cibelli@unifg.it (G.C.); chiara.porro@unifg.it (C.P.); giovanni.messina@unifg.it (G.M.); rita.polito@unifg.it (R.P.); 2 Department of Biosciences, Biotechnologies and Environment, University of Bari, 70125 Bari, Italy; antonia.cianciulli@uniba.it; 3 Department of Experimental Medicine, Section of Human Physiology and Unit of Dietetics and Sports Medicine, University of Campania “Luigi Vanvitelli”, 80138 Naples, Italy; vincenzo.monda@unicampania.it (V.M.); marcellino.monda@unicampania.it (M.M.); antonietta.messina@unicampania.it (A.M.)

**Keywords:** α-tocopherol, vitamin E, microglia, inflammation, central nervous system, neuroprotective effects, antioxidant action

## Abstract

Microglia, the resident macrophage-like population in the central nervous system, play a crucial role in the pathogenesis of many neurodegenerative disorders by triggering an inflammatory response that leads to neuronal death. Neuroprotective compounds to treat or prevent neurodegenerative diseases are a new field of study in modern medicine. Microglia are activated in response to inflammatory stimuli. The pathogenesis of various neurodegenerative diseases is closely related to the constant activation of microglia due to their fundamental role as a mediator of inflammation in the brain environment. α-Tocopherol, also known as vitamin E, is reported to possess potent neuroprotective effects. The goal of this study was to investigate the biological effects of vitamin E on BV2 microglial cells, as a possible neuroprotective and anti-inflammatory agent, following stimulation with lipopolysaccharide (LPS). The results showed that the pre-incubation of microglia with α-tocopherol can guarantee neuroprotective effects during microglial activation induced by LPS. α-Tocopherol preserved the branched morphology typical of microglia in a physiological state. It also reduced the migratory capacity; the production of pro-inflammatory and anti-inflammatory cytokines such as TNF-α and IL-10; and the activation of receptors such as TRL4 and CD40, which modulate the PI3K-Akt signaling pathway. The results of this study require further insights and research, but they present new scenarios for the application of vitamin E as an antioxidant for the purpose of greater neuroprotection in vivo for the prevention of possible neurodegenerative diseases.

## 1. Introduction

Vitamin E was first discovered by the American endocrinologist and anatomist Herbert M. Evans, together with his assistant Katherine S. Bishop [1]. The isolated substance, later named vitamin E, represents a family of lipid-soluble compounds consisting of four tocopherols and four tocotrienol derivatives. Each group can be further divided into four different isomers, that is, α, β, δ, and γ, depending on the presence and position of a methyl group(s) as a side chain. In general, vitamin E is formed by a chromanol ring and an isoprenoid or phytyl side chain. Tocopherols have a long and saturated side chain, while tocotrienols differ from tocopherols due to the presence of unsaturated double bonds on the side chain. This conformation explains the higher affinity of tocotrienols to the lipid membrane compared to tocopherols [2]. The definition of vitamin E and the derivates associated with this term are still under debate. While vitamin E is extensively used as an umbrella term for different tocopherol and tocotrienol forms, some authors have utilized it as a synonym for α-tocopherol. Azzi et al. justified the use of vitamin E as a synonym for α-tocopherol by considering the classification of vitamins, which describes them as a group of substances that are essential for normal metabolism, with deficiencies leading to disorders that are treatable by supplementation [3]. According to this definition, only α-tocopherol should be named vitamin E since it is the only form that has been shown to prevent the rare inherited neurodegenerative disorder ataxia with vitamin E deficiency (AVED), which is caused by mutations in the gene encoding α-TTP (alpha tocopherol transfer protein (TTPA)) [3,4]. However, since tocopherols and tocotrienols are generally both implied when using the term vitamin E in the scientific literature, we also use vitamin E as an umbrella term in this study [5]. Vitamin E, particularly α-tocopherol, can easily be obtained and consumed by most of the world’s population. Vitamin E isoforms are naturally present in various foods, mainly of vegetable origin, such as cereals, legumes, seeds, and seed oil [6], as well as in some foods of animal origin such as milk [7], dairy products [8], animal fats such as butter [9], egg yolk [10], and some fish products [11]. These types of foods are present in some diets, such as the ketogenic diet, which is rich in fats and proteins but low in carbohydrates and causes an effect similar to fasting by directing the body into a state of ketosis [12]. Vitamin E, in addition to being a fat-soluble compound [13], acts as an antioxidant, protecting the brain environment from oxidative stress through the ability to scavenge free radicals [14,15]. The central nervous system (CNS) has a high rate of oxygen consumption and contains a good amount of lipids. It is particularly sensitive to the possible damage caused by free radicals due to a relative functional insufficiency of the antioxidant systems compared to other tissues [16,17]. The cellular systems responsible for the defense from free radicals lose their effectiveness with the processes of normal cellular senescence or because of external factors, causing less defense, especially in the cerebral environment, which plays a fundamental role in neurodegenerative processes [18]. The role of vitamin E and its isoforms in the CNS have not been studied. In fact, there are few studies in the literature, despite vitamin E acting by protecting cell membranes from possible oxidative damage due to free radicals and hydrogen peroxide neutralizing its effects [19]. Furthermore, in addition to its antioxidant properties, vitamin E could also act as an anti-inflammatory agent with a consequent neuroprotective action [20]. As analyzed in a previous work [21], a neuroprotective action could be performed by vitamin E on microglial cells, the macrophages of the innate immune system present in the CNS and the main mediators of the neuroinflammatory process [20]. Microglial cells, in response to neuroinflammation or damage, adapt their morphology and functions by following signals from the brain environment [22]. These signals are important for various physiological functions, such as phagocytic and defense activity, the production of pro-inflammatory or anti-inflammatory cytokines, and the support of other brain cells [23]. Microglia, when activated by pro-inflammatory factors such as LPS, produce many pro-inflammatory factors, including pro-inflammatory cytokines such as tumor necrosis factor alpha (TNF-α), reactive oxygen species (ROS), and nitric oxide (NO) and increase the activation of the nuclear factor (NF-kB) [24] and PI3K/AKT [25] signal transduction pathways, resulting in increased expression of proteins such as CD40 [26], which are directly responsible for damage to the cells of the surrounding neuronal environment [27]. Recent studies have examined how treatment with antioxidants could have beneficial effects, especially at the level of microglia. In fact, different studies [21] have demonstrated how antioxidant treatments can reduce ROS, TNFα, IL-1β, and IL-6; reduce pro-inflammatory activation in LPS-stimulated macrophages in vitro; and increase the production of anti-inflammatory cytokines such as IL-10 [28]. Vitamin E has been shown to reduce the production of IL-1α and TNF-α, the expression of iNOS [29], and the activity of NFκB linked to stimulation with LPS after pretreatment with LPS [30,31,32]. Therefore, the present study aimed to investigate the biological effect of vitamin E on microglial cultures as a possible neuroprotective and anti-inflammatory agent following LPS stimulation in BV2 microglial cells.

## 2. Results

### 2.1. Influence of Vitamin E on BV2 Cell Viability

**Figure 1:**
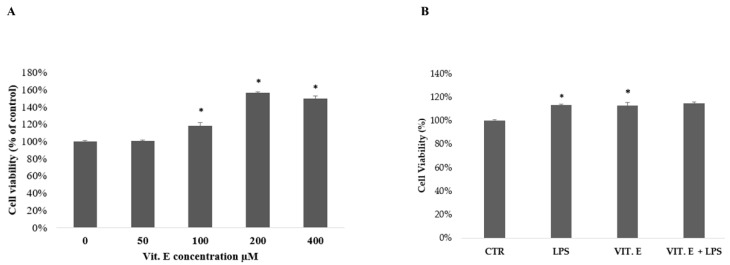
Cell viability analysis of vitamin E using an MTT assay. BV2 cells were treated with a vitamin E dose–response curve from 50 μM to 400 μM (**A**). Vitamin E at a concentration of 100 μM was used to treat cells in the absence or presence of 1 µg/mL LPS (**B**). Data are reported as percentages compared to control values and are expressed as means ± SDs. * *p* < 0.05 compared to the control of the same time point.

We tested different concentrations of vitamin E in BV2 cells in a dose–response curve using an MTT assay with concentrations ranging from 50 μM to 400 μM (Figure 1A). No cytotoxic effect was detected for any of the concentrations that were used. The lowest concentration that induced greater cellular viability was 100 μM, as also reported in some scientific studies [33]. This final concentration was chosen for the subsequent experimental tests. The MTT test was subsequently reproduced at 24 h with a vitamin E concentration of 100 μM in the presence or absence of LPS at a concentration of 1 µg/mL, as shown in Figure 1B.

As shown in Figure 1B, the treatment of BV2 microglial cells with vitamin E alone did not substantially change cell viability, unlike the LPS treatment, which induced an increase in cell viability after 24 h of treatment [34]. Furthermore, vitamin E co-administered with LPS did not show the ability to reverse the LPS-mediated increase in cell proliferation.

### 2.2. Vitamin E and BV2 Cell Morphology Changes

**Figure 2:**
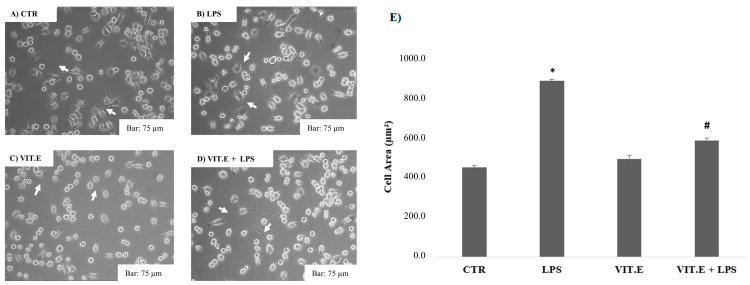
Morphological analysis after administration of vitamin E with or without LPS. Morphological analysis of BV2 cells in the control condition (**A**) and after 1 µg/mL LPS (**B**), 100 μM vitamin E (**C**), or 100 μM vitamin E in the presence of 1 µg/mL LPS (**D**). Bar: 75 µm (20 × objective). In the images, the arrows indicate cells that have undergone a morphological change. Cell areas (µm^2^) were quantified using ImageJ software bounded with Java8 64-bit (**E**). Data are expressed as the means ± SDs of the cell areas. * *p* < 0.05 compared to control. # *p* < 0.05 compared to LPS.

Since microglia are macrophages capable of modifying their morphology following extracellular signals [35], we performed morphological tests to evaluate how BV2 cell morphology was affected after the administration of vitamin E with or without LPS. The results are shown in Figure 2.

Figure 2 shows that untreated cells, corresponding to the control condition (A), had the classic morphology of microglia in a quiescent state, with a small central body and many elongated cell extensions. As expected based on current morphology studies [36], after treatment with LPS (B), the BV2 cells acquired an amoeboid morphology (linked to the pro-inflammatory condition), with increases in their somata and reductions in their prolongations, thus highlighting an amoeboid form. Treatment with vitamin E (C) did not affect the cell morphology compared to the control, thus showing a branched morphology with a small central soma (linked to the anti-inflammatory condition). Similarly, in the cells treated with vitamin E and LPS (1 µg/mL) (D), it was noted that vitamin E restored the amoeboid phenotype typical of LPS, causing greater branching of the distal branches. These results were also confirmed by an analysis of the cellular areas (E), which showed how the treatment with LPS increased the size of the BV2 cells, which is typical of the amoeboid form. In fact, they appeared to have significantly increased cellular areas compared to the control condition. Regarding the treatment of vitamin E in the presence of LPS (1 µg/mL) (E), the results showed that vitamin E can significantly reduce the increase in the cellular area induced by the LPS condition, inducing microglial cells to maintain their initial morphology, which is typical of the control condition.

### 2.3. Effect of Vitamin E on Cell Migration

**Figure 3:**
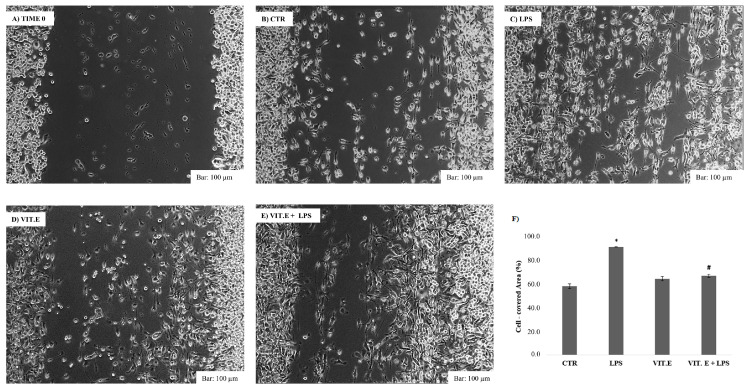
Analysis of the migratory capacity of microglia following the administration of vitamin E with or without LPS. A wound was procured from a sub-confluent layer of BV2 cells, and the resulting space was imaged at the time of the wound and 24 h after the treatment: BV2 cells at time 0 (**A**), 24 h after the cut in the control condition (**B**), with 1 µg/mL LPS (**C**), with 100 μM vitamin E (**D**), and with 100 μM vitamin E in the presence of 1 µg/mL LPS (**E**). The images are representative of an experiment with three independent replicates. The percentage of the wound gap was analyzed using ImageJ software and subsequently plotted and statistically analyzed as the percentage of wound closure compared to the 0 time condition (**F**). Values are presented as means ± SDs. Bar: 100 µm (10× objective). * *p* < 0.05 compared to control. # *p* < 0.05 compared to LPS condition.

To assess the influence of microglial motility following vitamin E treatment, we used a wound closure assay as a test, as described above. A cut was made with a scraper. The BV2 cells with the scratch created in the monolayer were allowed to migrate during a 24 h incubation. Consequently, the fixed and free cell areas were measured in order to understand whether the stimulus of vitamin E in the presence or absence of LPS induced wound closure. The results are shown in Figure 3.

The results show that LPS stimulation induced a greater cell migration potential than that of control cells after 24 h of incubation (C), significantly reducing the free wound surface after cutting. The application of vitamin E (D), as shown in Figure 3, did not result in major and significant motility of the BV2 cells, showing a free wound surface almost comparable to the control condition after 24 h (F), thus showing a migratory capacity markedly lower than in the LPS condition. Regarding the costimulation of vitamin E with LPS, Figure 3F shows that vitamin E caused a significant reversal of the LPS effect, leading to a significant reduction in microglial migration compared to BV2 cells treated only with LPS. Therefore, in both migration tests, vitamin E acted as a modulator of the migratory capacity by reducing BV2 cell motility, which was enhanced by the application of LPS.

### 2.4. Vitamin E Influenced Microglial Cytokine Concentrations

**Figure 4:**
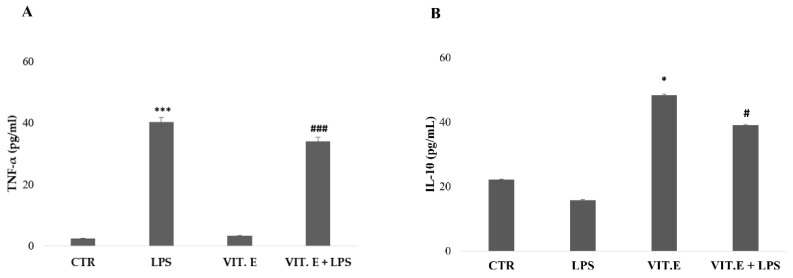
Evaluation of cytokine concentrations following administration of vitamin E with or without LPS. Analysis of cytokine concentrations (TNF-α (**A**) and IL-10 (**B**)) with stimulation of 100 μM vitamin E with or without LPS (1 µg/mL). Data are expressed as means (pg/mL) ± SDs. (**A**) *** *p* < 0.001 compared to the control condition. ### *p* < 0.001 compared to the condition of LPS-stimulated microglia. (**B**) * *p* < 0.05 compared to the CTR. # *p* < 0.05 compared to the LPS condition.

The anti-inflammatory effect of vitamin E on the concentrations of pro-inflammatory cytokines of BV2 cells was also evaluated. We analyzed the levels of TNF-α and IL-10. The results are presented in Figure 4.

As shown in Figure 4A, the concentration of TNF-α was significantly lower in the control condition and in cells treated with vitamin E, with almost overlapping results. In the condition of cells treated with LPS, the expression of TNF-α significantly increased compared to the control cells. BV2 cells treated with vitamin E + LPS showed a lower concentration of TNF-α that was significantly reduced compared to the LPS condition. At the same time, as shown in Figure 4B, the production of IL-10, an anti-inflammatory cytokine responsible for modulating and regulating the immune response, was significantly increased in vitamin-E-treated BV2 cells. Furthermore, the concentration of IL-10 in the vitamin E + LPS condition was significantly higher than in the LPS condition.

### 2.5. Effects of Vitamin E on the Expression of Pro-Inflammatory and Anti-Inflammatory Markers of Activated Microglia

**Figure 5:**
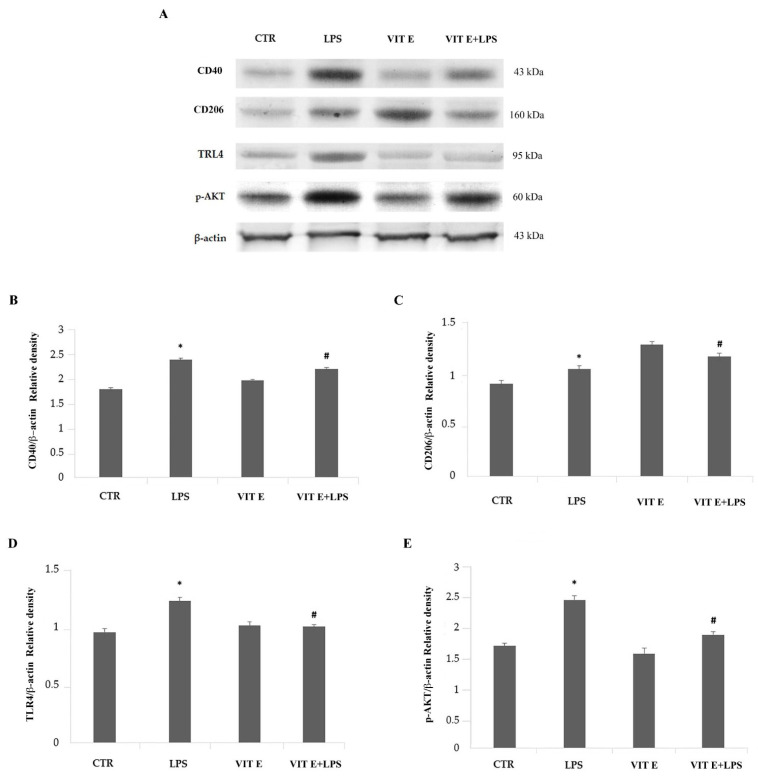
Evaluation of CD40, CD206, TLR4, and p-AKT expression following administration of vitamin E with or without LPS. Western blotting detection (**A**) and densitometric analysis of the expression of the pro-inflammatory CD40 (**B**), anti-inflammatory CD206 (**C**), TLR4 (**D**), and p-AKT (**E**) in control cells (CTR), BV2 cells treated with vitamin E (VIT E), BV2 cells treated with LPS (LPS), and BV2 cells treated with vitamin E + LPS (VIT E + LPS). Protein expression values are expressed in arbitrary units after normalization against β-actin. Data are presented as means ± SDs (* *p* < 0.05 vs. CTR; # *p* < 0.05 vs. LPS).

The expression levels of markers of the M1 phenotype (CD40) and the M2 phenotype (CD206) were determined using a Western blot analysis. The results, reported in Figure 5B,C, show that the LPS treatment of BV2 cells induced significant increases (*p* < 0.05) in the pro-inflammatory CD40 and the anti-inflammatory CD206 molecules in comparison to control. We next examined the changes in protein expression after the treatment with vitamin E.

Interestingly, a densitometric analysis of the immunoblotting bands revealed that vitamin E caused a significant reduction (*p* < 0.05) in the CD40 protein expression levels, indicating that this compound can negatively modulate this pro-inflammatory marker. Conversely, CD206 showed a significant increase (*p* < 0.05) in microglia in response to the vitamin E treatment in comparison to the cells stimulated with LPS alone, suggesting that the M2 phenotype may represent the predominant phenotype among BV2 cells (Figure 5).

Since the TLR4/Myd88 signaling pathway mediates the inflammatory response, leading to the production of pro-inflammatory cytokines, we evaluated whether the signal mediators TLR4 and p-AKT underwent a modulation in terms of protein expression levels. We found that the expression of TLR4 and p-AKT increased significantly in the presence of LPS (*p* < 0.05) compared to control. As shown in Figure 5A,B, the pre-treatment with vitamin E caused a significative downregulation of both TLR4 and p-AKT (*p* < 005) (Figure 5D,E), suggesting that vitamin E may act as a modulator of inflammatory responses targeting the TLR4/Myd88 signaling pathway.

## 3. Discussion

In this study, we investigated the biological effects of vitamin E on BV2 microglial cells stimulated with LPS.

The obtained results show that the pre-incubation of microglia with vitamin E can modulate the activation of BV2 cells in presence of LPS. In fact, vitamin E preserved the branched morphology typical of microglia in a physiological state and reduced the migratory capacity as well as the production of pro-inflammatory cytokines, such as TNF-α. Finally, we observed that the vitamin E treatment caused a significant reduction in the activation of typical pro-inflammatory receptors, such as TRL4 and CD40, which modulate the PI3K-Akt signaling pathway, thus suggesting a possible downregulation by vitamin E of the microglial pro-inflammatory responses. Although this topic has not yet been fully explored, in recent years scientific studies on antioxidants have been gaining more and more relevance in the scientific literature, especially in relation to neurodegenerative diseases. Among the best-known antioxidants, vitamin E was discovered in 1922 by the embryologist Herbert Evans and was initially considered as a factor capable of preventing fetal animal death [3]. Today, vitamin E is considered a real antioxidant [13] that plays a fundamental role in preventing oxidative stress by blocking the reactivity of free radicals, the main factors responsible for damage to cell membranes [37], and by donating hydrogen atoms and stabilizing them [38]. In the 1920s, vitamin E was utilized as a primary therapy in drug-resistant epilepsy [39,40].

Recent studies have demonstrated that the ketogenic diet (KD) is a cutting-edge dietary therapy that could have enormous potential for the treatment of neurodegenerative disorders such as Alzheimer’s disease [41,42], Parkinson’s disease [43], and amyotrophic lateral sclerosis [44]. It has long been known that brain cells are particularly susceptible to various kinds of dysfunctions [45] precisely because of their high functionality and the high demand for adenosine triphosphate (ATP). This intense activity leads the brain to consume oxygen (O_2_) at very high rates, leading to a proportionately high mitochondrial production of ROS [46]. When, due to genetic, external, or environmental factors, there is an imbalance in the regulatory systems of these functions, the presence of a persistent response could lead to inflammation in the brain [47], with consequent long-time neuronal damage and the subsequent appearance of neurodegenerative diseases [48]. One of the first cell lines involved in the modulation of the cerebral inflammatory response is microglia [49]. Microglial cells, commonly described as the “brain resident branched macrophages” [35], are the first cells involved in the defense and homeostasis of the brain environment [49]. Microglia have the ability to adapt their phenotype and functions in response to the brain environment itself and to certain stimuli; i.e., they can activate two different phenotypes: the pro-inflammatory M1 phenotype (classical activation), caused by stimuli such as LPS, interferon (IFN)-γ, and ROS, and the anti-inflammatory M2 phenotype (alternative or anti-inflammatory activation), predominantly mediated by anti-inflammatory factors such as IL-4 and IL-13 [50,51]. Following stimulus-mediated activations, microglial cells release many pro-inflammatory or anti-inflammatory factors, including pro-inflammatory cytokines such as TNF-α, IL-1β, and IL-6, with a concomitant increased production of nitric oxide (NO) and reactive oxygen species (ROS). In the case of anti-inflammatory activation, they release cytokines such as IL-10 and anti-inflammatory factors such as Arg-1 and IGF-1, which induce a reduction in inflammation and reproduce homeostasis in the brain environment [52]. Different nutraceutical foods can modulate microglial cells in the CNS [53,54]. The ketogenic diet appears to be one of the neuroprotective factors that modulates inflammation and excessive ROS production, modulating the expression of a receptor expressed mainly in microglia (HCA2) [55], dendritic cells, and macrophages [56], which allows the production of immunomodulatory metabolites [44] and the inhibition of NF-kB [57], with the consequent reduction and modulation of neuroinflammation [55]. This effect is assumed to also be mediated by antioxidants, especially vitamin E in this case, although there are still few studies in the literature. For this reason, considering the antioxidant and anti-inflammatory capacities of vitamin E, as described in the literature, in this study we examined its possible protective effects against LPS-induced toxicity in BV2 microglial cells. Our work showed that vitamin E does not seem to have a cytotoxic potential towards BV2 microglial cells, as it did not cause alterations in cell viability. Furthermore, it appears that vitamin E significantly suppressed LPS-induced microglial activation at the morphological and migratory levels and in terms of the pro-inflammatory cytokine production, as observed for TNF-α. Furthermore, vitamin E modulates the LPS-mediated activation of TLR4, with a consequent reduction in the CD40 molecule acting on the PI3K/AKT pathway. It is well known that LPS is an endotoxin. It is a component of the bacterial membrane [58] and has been described as a classical stimulus for microglial activation [59]. Basically, LPS binds to the TLR4 receptor, a receptor mainly expressed by microglial cells [60] that is responsible for the inflammatory cascade. Members of the TLR family play a key role as regulators of both the innate and adaptive immune responses [61], activating pro-inflammatory signals such as NF-κB and Akt [62] to induce the production of pro-inflammatory metabolites such as TNF-α, IL-6, and NO [63,64], with the subsequent maturation of antigen-presenting cells (APC) [65,66]. Furthermore, LPS induces the expression of the CD40 gene, which is a member of the TNFR family and is mainly expressed by cells such as macrophages, microglia, and dendritic cells as well as endothelial and tumor cells. The interaction between CD40 and its ligand is fundamental and is responsible for a pro-inflammatory immune response [67,68] with the production of pro-inflammatory cytokines and the enhancement of costimulatory molecule expression [69]. With our study, we provide results that allow us to affirm how vitamin E positively involves the suppression of basic signal transduction events involved in the activation of microglia, such as the reduction in TRL4 activation and the reduction in CD40 expression, resulting in the modulation of the PI3K/AKT pathway, with a consequent reduction in the expression of pro-inflammatory cytokines such as TNF-α, thus enhancing the idea of protection against a pro-inflammatory and potentially neurotoxic state, which is already supported by some studies in the literature [29,70]. Furthermore, as is known, microglia have migratory and phagocytic abilities [71,72], modulating the CNS microenvironment to respond to homeostatic changes [73] and at the same time causing a morphological change in the pro-inflammatory or anti-inflammatory phenotype [74]. The basic motility of microglia is characterized by the movement of typical cellular processes characterized by ramifications [75], which belong to the microglial morphology in the “resting state” [35]. The microglial morphology radically changes upon the detection of environmental stimuli in the CNS (pro-inflammatory foreign agents), such as LPS [76], or anti-inflammatory factors such as interleukin-4 (IL-4) or interleukin-13 (IL-13). Microglia are rapidly activated. The pro-inflammatory phenotype is characterized by the retraction of branching and migration processes towards tissue damage or a site of injury [77,78,79], with a typical change to the M1 pro-inflammatory morphological type [74] to secrete cytotoxic substances such as cytokines [80] and TNF-α [81]. In the case of alternative activation, the M2 phenotype is characterized by branched cellular processes and reduced cytoplasm, showing a reduced migratory capacity [82], and the subsequent production of anti-inflammatory cytokines such as interleukin-10 (IL-10) or positive factors for tissue repair and reconstruction of the extracellular cellular matrix (ECM) [83,84]. Although our results did not show a strong ability to upregulate the expression of CD206, a typical anti-inflammatory marker, its reduction was on the verge of significance, suggesting a more favorable action for the alternative response of microglia in the vitamin E treatment. Therefore, if it is true that antioxidants reduce inflammatory processes, vitamin E should also be characterized by the ability to reduce the migration of BV2 microglial cells and the consequent phenotypic change from the activated form to the resting form. Thus, the result that vitamin E plays a neuroprotective role by modulating microglial functions opens possible future scenarios for its therapeutic use for neurodegenerative diseases. A limitation of this research is represented by the in vitro model of immortalized cells. Further investigations using primary cultures or animal models are needed to confirm these observations.

## 4. Materials and Methods

### 4.1. Primary Microglial Cell Culture and Treatments

A BV2 murine microglial cell line was used in this study. The cells were purchased from the American Type Culture Collection (Manassas, VA, USA). BV2 cells were cultured in Dulbecco’s Modified Eagle Medium (DMEM) supplemented with 10% fetal bovine serum (FBS; Euroclone; Milan, Italy), 100 units/mL penicillin, 100 μg/mL streptomycin, (Penicillin-Streptomycin; Euroclone; Milan, Italy) and 2 mM glutamine (Glutamine; Euroclone; Milan, Italy) at 37 °C in a humidified incubator with a CO_2_ level equal to 5%. For subsequent experiments, BV2 cells were plated at an appropriate number and density after detaching them from the substrate using Trypsin-EDTA We have adde this information(Trypsin-EDTA 1X in PBS, Euroclone, Milano, Italy). The day after plating, two groups of cells were treated with 100 μM vitamin E and 1 μg/mL LPS (lipopolysaccharides from *Escherichia coli* O128: B12; Sigma-Aldrich, St. Louis, MI, USA), respectively, and another one was treated with vitamin E and LPS after 1 h. The cells were allowed to grow and were collected from the treatments after 24 h.

### 4.2. Preparation of Vitamin E Solution

Vitamin E (DL-α-tocopherol acetate ≥96% (HPLC); Sigma-Aldrich; CAS: 7695-91-2) was initially obtained by creating a solution with 1 M concentration diluted in ethanol (absolute ethanol; Scharlau). For the following experimental tests, the final concentrations were created, starting from the stock solution, by diluting the vitamin E in DMEM. Prior to the experiments, cells were incubated in DMEM containing vitamin E for up to 24 h [85].

### 4.3. Cell Viability Assay

The cytotoxicity of vitamin E was assessed using an MTT assay with Thiazolyl Blue Tetrazolium Bromide purchased from Sigma-Aldrich (CAS: 298-93-1). For the MTT assay, the BV2 cells were plated in 24-well plates with a density of 2 × 10^5^ cells and were incubated at 37 °C with 5% CO_2_ for 24 h with initial vitamin E concentrations of 50 μM, 100 μM, 200 μM, and 400 μM in order to study the cytotoxicity of vitamin E. Subsequently, we utilized the lowest concentration that induced an effect to evaluate the possible protective effect of vitamin E. The MTT assay was performed with 100 μM vitamin E in the presence or absence of LPS at a concentration of 1 µg/mL for 24 h. To read the absorbance, a spectrophotometer (Filter Max F5 Multi-Mode Microplate Reader, Molecular Devices, San Jose, CA 95134, USA) with a wavelength of 595 nm was used. The results are shown as the cell viability (%) based on the control condition.

### 4.4. Cell Morphology Analysis

An analysis of microglial morphology was carried out by means of a morphological image test to evaluate the effect of vitamin E with a concentration of 100 μM in the absence or presence of the pro-inflammatory stimulus (LPS with a concentration of 1 µg/mL). About 5 × 10^5^ cells were plated on a 6-well plate. All morphological tests were performed in triplicate, and the results are expressed as the average of the areas of three cells from five independent experiments.

The plates were evaluated using Leica Microscopy photography (DM IRB Leica Microsystems GmbH, Wetzlar, Germany) at 10× and 20× magnifications. The cell areas (µm^2^) were quantified using ImageJ software.

### 4.5. Cell Wound Closure Assay

To assess cell migration, we used a cell wound closure test. A total of 1 × 10^6^ BV2 cells were added to the wells on a 6-well plate. The cells were cultured until confluence. Confluent monolayers were injured with a sterile scratch using a scraper; subsequently, after washing with PBS and changing the DMEM, the remaining cells were incubated for 24 h overnight under different conditions with 100 μM vitamin E in the absence or presence of LPS with a concentration of 1 µg/mL. All migration tests were performed in triplicate. Closure of the open scar was documented after 24 h with photomicrographs of the various conditions that were analyzed. Wound closures were analyzed using ImageJ software and are expressed as a percentage of the cell-covered area from the zero-time condition.

### 4.6. Cytokine Determination

Vitamin E, at a concentration of 100 μM in the absence or presence of LPS (1 µg/mL), was added to BV2 cells and incubated at 37 °C with 5% CO_2_ for 24 h. After 24 h, the culture medium was collected and used for the evaluation of pro-inflammatory cytokines, such as TNF-α, and anti-inflammatory cytokines, such as IL-10, using commercially available ELISA kits (R&D Systems a bio-techne brand). The protocols used for the procedures were in accordance with the manufacturers’ instructions. The cytokine concentrations (pg/mL) in the medium were determined by referring to standard curves obtained with known amounts of TNF-α and IL-10.

### 4.7. Western Blotting

After previously described cell treatments, BV2 cells were detached from the plate by scraping and were collected after centrifugation at 2000 rpm for 10 min at 4 °C. The cells were lysed with an ice-cold lysis buffer composed of Tris-HCl (50 mM) at PH 8, 1% (*v*/*v*) Triton X-100, 1.5 M NaCl, 0.1% SDS, 100 μM phenylmethylsulfonyl fluoride (PMSF), 1 μM leupeptin hemisulfate salt, and 4 U/mL aprotinin (all from Sigma Aldrich). After eight cycles of freezing and thawing, the lysates were obtained by centrifugation at 12,000 rpm for 30 min at 4 °C. The concentrations of the lysates were determined using the Bradford assay, and for each treatment the same amount of protein (20 µg) was subjected to SDS-PAGE (NuPage Electrophoresis System, Invitrogen) mixed with NuPage LDS Sample Buffer (4×, 1:4 (*v*/*v*)) and NuPage Sample Reducing Agent (10×, 1:10) on 4–12% Novex Bis-Tris Midi gel 1.0 mm precast gels (Life Technologies Van Allen Way, Carlsbad, CA 92008, USA). At the end of the electrophoretic run, resolved proteins were transferred from the gel to a nitrocellulose membrane using iBlot Dry Blotting System A (Life-Technologies). Membranes, after blocking with 5% (*w*/*v*) non-fat dried milk for 1 h, were washed 3 times with 0.1% Tween 20-PBS (T-PBS). Primary antibodies directed against β-actin (1:500), CD40 (1:500), CD206 (1:500), TLR4 (1:500), and p-Akt (1:500), all obtained from Santa Cruz Biotechnology, Inc. (Santa Cruz, Heidelberg, Germany), were incubated for 1 h at room temperature on a shaker and then overnight at 4 °C. Next, the membranes were incubated with horseradish peroxidase (HRP)-conjugated secondary antibodies (Santa Cruz Biotechnology, diluted 1:10,000) for 60 min at room temperature in the dark on a shaker. After three washes with 0.1% Tween 20-PBS (T-PBS), immunoreactive bands were visualized using chemiluminescence (BioRad Laboratories, Hercules, CA, USA). Lastly, β-actin was used as a housekeeping protein to normalize protein expression levels [32]. The bands obtained after immunoblotting were submitted to a densitometric analysis using the ImageJ platform, and the results are expressed in arbitrary units.

### 4.8. Statistical Analysis

Statistical analyses were performed via two-sample comparison, analysis of variance (one-way ANOVA), and Tukey’s post hoc test using Statgraphics Centurion (Statgraphics Technologies Inc., The Plains, VA, USA). Values of *p* < 0.05 were considered statistically significant. All the assays were performed in triplicate, and the data are expressed as the means ± SDs of five independent experiments.

## 5. Conclusions

Our results highlight, for the first time, an interesting aspect of vitamin E. In the presence of a pro-inflammatory stimulus, vitamin E is able to modulate the microglial responses from a morpho-functional point of view by reducing microglial migratory properties and influencing the expression of the typical pro-inflammatory markers. Furthermore, vitamin E has been shown to be able to inhibit the activation of molecules and pro-inflammatory patterns by reducing the production of typical secondary metabolites of the neuroinflammatory response. The results require further scientific research, but for these effects, vitamin E seems to have potential and efficacy and could be used in the prevention and management of neurodegenerative diseases, suggesting its use as a dietary supplement for the treatment of neuroinflammation.

## Data Availability

Data sharing is not applicable to this article.
